# Current Trends in Mucosal Melanomas: An Overview

**DOI:** 10.3390/cancers15051356

**Published:** 2023-02-21

**Authors:** Davide Adriano Santeufemia, Giuseppe Palmieri, Gianmaria Miolo, Maria Colombino, Maria Grazia Doro, Laura Frogheri, Panagiotis Paliogiannis, Giampiero Capobianco, Massimo Madonia, Antonio Cossu, Giovanni Lo Re, Giuseppe Corona

**Affiliations:** 1 Oncology Unit, Alghero General Hospital, 07041 Alghero, Italy; 2 Immuno-Oncology & Targeted Cancer Biotherapies, Unit of Cancer Genetics, University of SassariIRGB-CNR, 07100 Sassari, Italy; 3 Preventive Medical Oncology, CRO Aviano, National Cancer Institute, IRCCS, 33081 Aviano, Italy; 4 Institute of Genetic and Biomedical Research (IRGB), National Research Council (CNR), 07100 Sassari, Italy; 5 Department of Medical, Surgical and Experimental Sciences, University of Sassari, 07100 Sassari, Italy; 6 Department of Clinical and Experimental Medicine, Gynecologic and Obstetric Clinic, University of Sassari, 07100 Sassari, Italy; 7 Department of Clinical and Experimental Medicine, Urologic Clinic, University of Sassari, 07100 Sassari, Italy; 8 Immuno-Related Tumor Medical Oncology, CRO Aviano, National Cancer Institute, IRCCS, 33081 Aviano, Italy; 9 Immunopathology and Cancer Biomarkers, CRO Aviano, National Cancer Institute, IRCCS, 33081 Aviano, Italy

**Keywords:** cancer, mucosal melanomas, diagnosis, prognosis, genetic, metabolism, immunotherapy

## Abstract

**Simple Summary:**

This review provides an updated overview about molecular features, clinical advancements and therapeutic directions of the primary mucosal melanoma which represents a rare and heterogeneous malignancy characterized by a poor prognosis.

**Abstract:**

Primary mucosal melanomas (MMs) are uncommon tumors originating from melanocytes located in the mucous membranes at various anatomic sites within the body. MM significantly differs from cutaneous melanoma (CM) regarding epidemiology, genetic profile, clinical presentation, and response to therapies. Despite these differences, that have important implications for both disease diagnosis and prognosis, MMs are usually treated in the same way as CM but exhibit a lower response rate to immunotherapy leading to a poorer survival rate. Furthermore, a high inter-patient variability can be observed in relation to therapeutic response. Recently, novel “*omics*” techniques have evidenced that MM lesions have different genomic, molecular, and metabolic landscapes as compared with CM lesions, thus explaining the heterogeneity of the response. Such specific molecular aspects might be useful to identify new biomarkers aimed at improving the diagnosis and selection of MM patients who could benefit from immunotherapy or targeted therapy. In this review, we have focused on relevant molecular and clinical advancements for the different MM subtypes in order to describe the updated knowledge relating to main diagnostic, clinical, and therapeutic implications as well as to provide hints on likely future directions.

## 1. Introduction

Primary Mucosal Melanoma (MM) is a rare malignancy originating from melanocytes located in the mucosae at various anatomic sites within the body [1]. Estimation of cancer incidence in United States for 2023 indicates that melanomas will represent 5% of new cancer cases, of which MM will constitute ≤2% diagnoses [2].

Melanocytes’ primary role is the synthesis of the melanin pigment that, in the skin, is transferred to keratinocytes and serves as a shield to protect the human body surface from sunlight UV-radiation. Despite the fact that these pigmented cells are markedly distributed into the skin and the vast majority of melanomas are of cutaneous origin [2,3], they can also be found in other tissues. Originating from pluripotent embryonic cells (namely, the neural crest cells), melanocytes have the property to migrate not only under the epidermis to populate all parts of the skin but also within the basal cell layer of the epithelium at different anatomic districts. Melanocytes are mainly present in the inner ear, nervous system, and heart; in addition, generally they can be found in nearly all tissues to a differing degree [4].

Melanin-containing melanocytes are present in the basal cell layer of the epithelium even at mucosal sites with no visible signs of melanin pigmentation [5,6,7]. At the oral level, melanocytes may or may not produce melanin and, analogously to what happens into the skin, the amount of synthetized melanin is constitutionally determined [3,7]. As is commonly known, there are substantial variations in rates of melanin pigmentation among people from different racial/ethnic groups as well as among those belonging to the same racial/ethnic group [3,8,9]. Furthermore, when considering the oral site, melanin pigmentation is common in black people [10], being even more frequent in darker skinned whites (Caucasians) than in lighter skinned whites [11]. Additionally, melanin distribution into the oral mucosae may be heterogeneous, with the pigmentation being patchy or uniform and more frequently affecting the gingiva [10]. Smaller amounts of melanin-containing melanocytes can be also found in other distinct mucosal membranes, determining that melanomas can arise in any site where such cells are present: the respiratory, gastrointestinal, and urogenital tracts as well as in the eye [3,11].

MM significantly differs from cutaneous melanoma (CM) regarding epidemiology, genetic profile, clinical presentation, and response to therapies [5,6]. Despite these differences that have important implications for disease management, the MMs are usually treated in the same way as CMs [6]. However, MM patients exhibit a lower response rate to immunotherapy than CM patients, with a poorer survival rate [5,6,7]. Furthermore, a high inter-patient variability can be observed in relation to treatment response.

Recently, novel omics’ techniques have revealed that MMs have a different molecular landscape and genomic profiling as compared with CM that can explain their different response [8,12]. Such specific molecular aspects are useful for identifying new biomarkers for improving diagnosis as well as the selection of MM patient candidates to achieve the best benefit from immunotherapy or targeted therapy.

Here, we will we provide an updated overview of the relevant molecular and clinical advancements on MM subtypes in order to describe the state of the art on major diagnostic, clinical, and therapeutic implications as well as likely future directions.

## 2. Mucosal Melanomas

### 2.1. Epidemiology

MMs are very rare tumors representing less than 2% of all melanomas [8,9], with about 850 new cases reported in Europe in 2013 [6]. Unlike CM, whose incidence is increasing, the MM incidence is estimated to remain stable [2,10,11,13,14]. Usually, MMs are diagnosed in people aged over 65 years, with a rate of 6.3 per million, and they are more frequent in women than men [6].

MMs occur with about equal frequency in all races, therefore accounting of a substantial fraction of all melanomas diagnosed in black people, Asian people, and Hispanic people when compared with melanomas occurring amongst Caucasian people [15]. This reflects the incidence of CM in these populations [15].

Defined as melanomas occurring in mucosal membranes at various anatomic sites, these lesions are most frequently observed in the head and neck regions (approximately 50% of cases) followed by gastrointestinal (37%), female genital (6%), and urinary tracts (4%) rather than other locations such as pharynx, larynx, urinary tract, cervix, esophagus, and gallbladder [16]. Furthermore, lung site involvement is mostly secondary [17].

### 2.2. Risk Factors

Unlike CM, where association with UV sunlight exposure represents a well-defined risk factor, genomic studies have suggested that UV light plays a nearly null role in MM carcinogenesis [8,12,18,19]. In fact, MMs develop on sun-shielded surfaces.

Risk factors for this rare melanoma subtype remain poorly understood. Currently, there is no clear evidence to support the pathogenic role of chemical carcinogens or viruses despite their suggestion as etiologic factors [15].

Interestingly, some authors have reported that oral melanosis, which precede about one third of MMs in the oral cavity, may be associated with cigarette smoking [20,21].

### 2.3. Molecular Features

Globally, MMs are characterized by the presence of distinct molecular features, frequently characterized by DNA structural changes and mutation signatures of unknown etiology, with respect to CM [22,23].

MMs are characterized by a relatively low tumor mutational burden (TMB) compared to CM [24]. In particular, MM has an average of about 8000 non-synonymous single nucleotide variants (nsSNVs) per tumor, a more than 10-fold lower mutation rate than CM (which, with an average of more than 80,000 nsSNVs, exhibits one of the highest somatic neoplastic mutation rates) [8,24,25]. This difference may explain the molecular reason for discordant clinical responses to immunotherapy that are observed in Asian people, where MM represents a substantial fraction of all diagnosed melanomas, versus white Caucasian people [15,26,27].

From the qualitative point of view, mutational profiling of MM as inferred by whole genome/exome sequencing approaches is different from that observed in CM [28]. According to recent NGS-based studies as well as meta-analysis and systematic review, MMs are characterized by a lower load of somatic mutations and lack of either UV-induced mutational signatures or oncogenic fusion gene transcripts frequently reported in CM [29,30,31].

**Figure 1:**
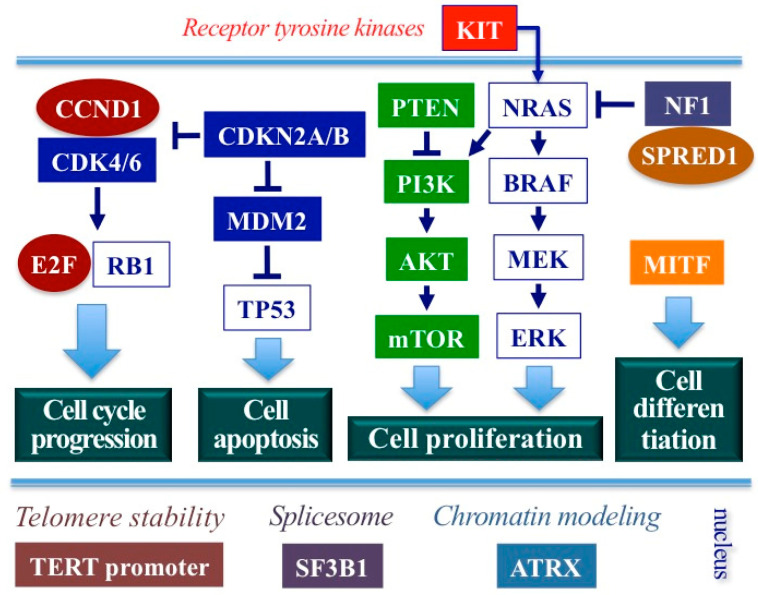
Main genes and their functional role in MM. ATRX, ATP-dependent helicase; BRAF, B-Raf proto-oncogene; CDK4/6, Cyclin-dependent kinase 4/6; CCND1, Cyclin D1; CDKN2A/B, Cyclin-dependent kinase inhibitor of kinase 2A/B; E2F, E2F transcription factor 2; ERK, Extracellular-related kinase; KIT, receptor tyrosine kinase; MDM2, mouse double minute 2 p53 binding protein homolog; MITF, Microphthalmia-associated transcription factor; MEK, Mitogen-activated protein kinase-extracellular related kinase; mTOR, mammalian target of rapamycin; NF1, neurofibromin 1; NRAS, neuroblastoma RAS viral oncogene homolog; PI3K, Phosphatidylinositol 3 kinase; PTEN, Phosphatase and tensin homologue; RB1, retinoblastoma 1; SF3B1, splicing factor 3b subunit 1; SPRED1, sprout-related EVH1 domain containing protein 1; TERT, telomerase reverse transcriptase; TP53, tumor protein p53.

In Figure 1, the main genes and their functional pathways involved in MM pathogenesis are represented. Overall, the genes mostly mutated (>10%) are neurofibromin 1 (*NF1*), KIT proto-oncogene receptor tyrosine kinase (*KIT*), and splicing factor 3b subunit 1 (*SF3B1*); the remaining genes commonly mutated but at lower rates (5% to 10%) are neuroblastoma RAS viral oncogene homolog (*NRAS*), sprout-related EVH1 domain containing protein 1 (*SPRED1*), ATP dependent helicase (*ATRX*), and B-Raf proto-oncogene (*BRAF*) [29,30,31]. The MITF gene, involved in melanocyte development and differentiation, has been found to either rarely carry the germline variant E318K—widely demonstrated to confer an increased CM risk—in vulvar melanoma either to be commonly amplified in several MMs [30,32].

**Figure 2:**
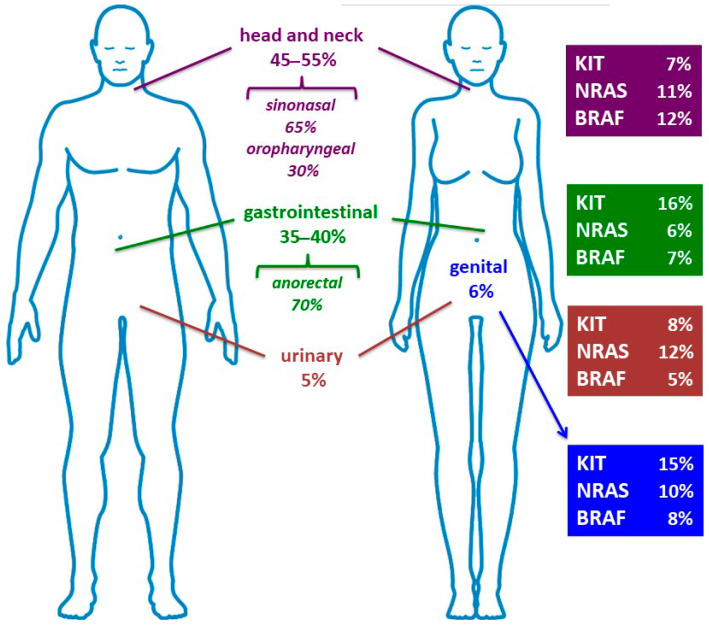
Distribution of MM anatomical locations (head and neck, gastrointestinal, urinary, and genital) and correspondent mutation frequencies for the main driver oncogenes (BRAF, NRAS, and KIT). Mutation prevalence values were mainly derived by NGS-based approaches (for references, see text).

Despite the fact that the *BRAF* and *NRAS* genes are preponderantly mutated in CM lesions (together accounting for about 75% of cases), their role in MM pathogenesis is not secondary. Activating mutations of the *BRAF* oncogene are detected in approximately 50% of CM [28], but at much lower frequency in MMs [13,14,19,33,34,35]. Considering NGS-based data, the frequency of mutations for *BRAF* and *NRAS* oncogenes has been reported at even higher rates (up to more than 20%) in different types of MM, with the most prevalent incidence in the mucosae of the head and neck district (Figure 2) [29,30,31].

In sinonasal MM, a high prevalence of *NRAS* mutations has been reported [36]; however, a lack of *KIT* alterations with a high prevalence of *BRAF* mutations has been reported in some populations, such as those from South Italy [13] or from Germany [37], but not in the majority of the others (as those from Japan, Poland, Spain, and the United States [38]), further suggesting that patients’ origin may account for different mutation rates in candidate cancer genes. In some MM cases with a lower rate of *BRAF* mutations, an increased activity of the Cyclin D1-Cyclin-dependent kinase 4/6 (CCDN1-CDK4/6) complex, mostly through gene amplification, has been demonstrated as contributing to MM pathogenesis [39]. Finally, in *BRAF*/*NRAS*-wild-type MMs, a higher rate of mutation into the Telomerase Reverse Transcriptase (*TERT*) promoter has been described, specifically for sinonasal melanomas [29,36]. Not having UV radiation as the most preponderant carcinogenic effect—usually occurring in the CM etiology—the pathogenic sequence variations found in the *BRAF* and *NRAS* genes appear to be more heterogeneous in MMs [33]. In fact, a markedly high prevalence of mutations at the V600 codon of *BRAF* (nearly all BRAF-mutated CM lesions present V600E/K variants, with particular reference to melanomas arising on skin areas intermittently exposed to UV) is lacking among MM cases; conversely, sequence variations mostly affect other regions of the *BRAF* gene than codon 600, though most of them in codons of the exons 11 and 15 are deputed to encode the protein domains where the kinase activity resides (among others, codons D594, G469, and K601 are frequently altered) [22,33,40,41].

This situation closely resembles what occurs in the adenocarcinoma subtype in the non-small cell lung cancer (NSCLC), in which the V600E mutations account for a fraction of BRAF-mutated NSCLC cases (about 40%, mainly females, and never-smoker patients), whereas the majority of them (about two thirds, regardless of the smoking habitus) carry non-V600 mutations [42]. Similarly, a poor prevalence of mutations into the Q61 codon of the *NRAS* exon 3, which is found in nearly all RAS-mutated CM cases, has been observed in MM; conversely, the G12 and G13 codons within the exon 2 of the gene are maximally involved in NRAS-mutated MM cases [29,30,31]. The latter codons are also prevalently mutated in the *KRAS* gene among lung and colorectal adenocarcinomas [43]. Such evidence strongly supports the hypothesis that causative factors promoting mutations in *BRAF* and *NRAS* genes in MM can be more similar to those involved in the onset and progression of lung and colorectal carcinomas than to those involved in cutaneous melanocytic transformation. Unfortunately, such causative factors remain to be determined.

As already depicted above, the anatomical sites where MMs originate deeply affect the spectrum and distribution of the mutations in driver genes (Figure 2). Considering the main one, head and neck melanomas present a lower prevalence of *KIT* mutations as compared to the genitourinary tract and anorectal melanomas in which mutations and amplifications of this gene are common; among genital MMs, vaginal but not vulvar melanomas present very low rates of *KIT* mutations [40,41,44,45,46,47].

Furthermore, the frequency of different mutations may vary across different studies and reports on patients of different geographical origin and different genetically driven constitutional features. For other genes, a different anatomical distribution of alterations has also been reported: *ATRX* and *SF3B1* mutations occurred more commonly in lower anatomy MMs and *TERT* or *CTNNB1* in the upper anatomy [30]. Overall, MMs show specific genetic signatures depending on the site of origin. A higher percentage of mutations involving the *SF3B1* gene was observed in patients with MM of anorectal origin. In patients with vulvovaginal melanoma, the involvement of the *TP53* gene is common, while mutations involving the *MYC* gene are frequently detected in patients with sinus or nasopharyngeal melanoma [30,31,47].

In summary, the mitogen-activated protein kinase (MAPK) pathway—including the RAS-RAF-MEK-ERK signal transduction cascade—regulates the main processes of cell proliferation and cell survival in both MM and CM lesions. However, while in CM the MAPK cascade is directly activated by a constitutive alteration of one of its component (*BRAF* or *NRAS*), in MM the oncogenic MAPK activation is indirectly promoted by effectors external to the pathway (*KIT* and/or *NF1*) (Figure 1). In particular, *KIT* encodes a tyrosine kinase receptor of the cell membrane and its activation through mutation and/or amplification result in a continuous RAS-driven induction of cell proliferation, whereas *NF1* encodes for a negative regulator of RAS signaling and its genetic or functional silencing results in RAS activation and enhancement of the malignant transformation and progression [48]. Inactivation or functional loss of *NF1* may also cooperate with other so-called RASopathy genes—such as *SPRED1*, which is involved in melanoma genesis (Figure 1)—in sustaining constitutive RAS activation [48]. Tumors with mutations in *NF1* and constitutive activation of RASopathy genes are expected to be associated with a higher mutational load and, consequently, a greater probability of generating neoantigens [49,50]. Unfortunately, MMs are poorly immunogenic, regardless of their intracellular mutational status, probably due to their lack of ability to induce a strong adaptive immune response in the tumor microenvironment; the reduced susceptibility to PD-1/PD-L1 and/or CTLA-4 blockade is highlighted by the extremely poor clinical outcomes of patients with advanced MM using immune checkpoint inhibitors [51].

### 2.4. Clinical Behavior

Globally, MMs are characterized by an aggressive clinical behavior and a prognosis worse than CM [40]. Early diagnosis of the disease can be hindered by both its rarity and its mostly occult anatomical origin site; a possible multifocality; and/or the fact that about half of MMs are amelanotic may contribute to delaying diagnosis [41,52].

MMs patients are most likely to be diagnosed with regional or distant metastases, with a reported 5-year overall survival rate of approximately 25% [9,40]. In the large series reported by Lian et al. [40], predominant metastatic sites included regional lymph nodes (21.5%), lung (21%), liver (18.5%), and distant nodes (9%).

Interestingly, when matched for staging, prognostic, and molecular factors, no significant survival difference among MMs arising at different sites was found by Cui et al. in a series of 706 patients [53].

## 3. Clinical Presentation

MM clinical presentation is extremely variable. The site of origin and the size of the tumor, along with the possible metastatic involvement of other locations within the human body, can clearly determine different clinical scenarios.

### 3.1. Head and Neck

Although almost all malignant melanomas of the head and neck district are constituted by cutaneous lesions, mostly arising on the face skin, this region represents the most common site of MM origin and it accounts for more than half of all diagnosed MMs [10,54].

MMs may involve the nose and paranasal sinuses, oral cavity, pharynx, larynx, and esophagus [1]. The nose and paranasal sinuses followed by the oral cavity are the most common sites of origin [54,55], whereas it is uncommon to find it in the primitive mucosa of the larynx, pharynx, esophagus, or tracheobronchial tree [1,17,54,56,57,58].

MMs arising from the respiratory mucosa (such as the nasal cavity) have different clinicopathologic characteristics than those involving the oral mucosa, though they are characterized by similar adverse prognosis and outcome [54].

Within the nasal cavity, the origin of the disease from either the lateral nasal wall or nasal septum is preferential, whereas, considering the sinuses, the maxillary sinus, followed by the ethmoid, the frontal, and the sphenoid sinuses, these represent the anatomic privileged sites of its origin [54,59].

Typically, most of patients developing a MM in the nasal cavity presents a clinically localized disease and tend to exhibit a more favorable prognosis than those affected by MMs of paranasal sinuses or of other head and neck sites, which are often diagnosed at a more advanced disease stage with subsequent poorer outcome [54,59]. Sinus melanomas are asymptomatic in this anatomically silent area until the neoplastic growth invasion of adjacent structures is evident. In a pooled data analysis of approximately 200 patients from five series, the five-year survival for patients with nasal melanoma was 31% compared to approximately 0% for those with sinus melanoma [1,54,60].

Sinonasal MM commonly metastasizes to the lung and liver. At the time of the diagnosis, nodal secondary involvement by the tumor is present in about 20% of patients [54,60]. Clinical symptoms—such as epistaxis, unilateral nasal obstruction, or persistent rhinorrhea, loss of smell, and facial pain—are nonspecific, often indistinguishable from those of benign sinonasal disease; therefore, early diagnosis can be delayed in several cases [59]. Moreover, proptosis, diplopia, or neurological symptoms are symptomatic of a more advanced disease stage [60].

Most of such MMs is likely to show features endoscopically as polypoid, fleshy lesions with strict unilateral involvement with different degrees of pigmentation while they are rarely amelanotic [6].

Oral cavity MMs represent almost 30% of MMs of the head and neck, mostly involving the palate and maxillary gingiva [60,61]. Again, they are asymptomatic in the early stages, usually presenting with a pigmented patch or a mass characterized by a rapid growth rate [62].

Diagnosis may be easier due to greater accessibility of the oral cavity for clinical examination. Those lesions can be amelanocitic or characterized by a wide range of colors varying from black, brown, grey, to reddish or white [6,63].

At the time of the diagnosis, the probability of detecting distant metastases is low (5–10%) [6] with no significant difference between oral and sinonasal MMs; however, the incidence of nodal metastases at this level is highest than in sinonasal lesions, especially in presence of a depth of infiltration > 5 mm [6,61].

### 3.2. Gastrointestinal MMs

Approximately, 37% of MMs arise from the gastrointestinal tract, which represents the second most privileged site of origin for those malignancies [16]. Their preferred location is the anorectal tract (>50%), followed by the stomach, small intestine, and colon [64].

However, anorectal MMs are very rare accounting for approximately 0.5% of all colorectal and anal cancers [65]. MM rectal origin is more frequent than anal primary involvement and notably, given their infrequent occurrence, those tumors are studied together as a unique entity [64]. They are most frequent in women aged over 50 years and are characterized by a very poor prognosis [66]. It is also interesting to report that there is an unclear association between HIV infection and anorectal MM, though we think it noteworthy to take this information into account in the management of such patients [66].

Because of the hidden location and the lack of specific symptoms, patients are likely to present with disseminated disease [66]. Typical, early symptoms such as itching or rectal pain are often mistakenly attributed to a benign pathology, such as hemorrhoids, polyps, or anal fissures, and so the diagnosis is delayed many times. Moreover, changes in bowel habits, bowel obstruction, rectal bleeding, anal pain, and/or rectal tenesmus occur in a more advanced disease stage.

### 3.3. Genitourinary MMs

MM arising from the genitourinary (GU) tract is an extremely rare disease, characterized by a clinically aggressive behavior, that comprises 0.2–1% of all melanoma cases [64]. GU MMs include tumors arising from the female GU tract (vulva, vagina, and cervix), male GU tract (penis and scrotum), and urinary tract (urethral and bladder) [64].

In a series of 817 primary GU melanoma cases from the Surveillance, Epidemiology, and End Results (SEER) database, the female GU tract was the most commonly involved site (89.4%), followed by the male GU tract (6.6%), and the urinary tract (4.3%) [64,67]. Most cases of GU MMs occurred in the vulva, with the highest age-specific incidence rates in patients aged 85 years and older for both women and men [67]. The labia minora followed by the labia majora and clitoris represent the most common sites of disease occurrence, whereas urethra and cervix locations are less frequently involved [68]. Symptoms of vulvovaginal melanoma include itching, vaginal discharge, bleeding, dyspareunia, mass as well as occasional dysuria and voiding dysfunction if urethral involvement occurs [69].

Vulvovaginal melanomas present frequently with regional lymph node or distant metastatic disease, probably due to the richly innervated lymphatic system that facilitates nodal spreading; again, their prognosis is very poor [64].

GU melanoma usually presents in males as a pigmented macule, papule, plaque, or an irregularly demarcated, ulcerated lesion on the glans penis at an early disease stage. Moreover, patients may present obstructive symptoms, hematuria, urethral discharge, and urinary fistula in more advanced disease stages [64]. Prognosis is also very poor. Hematuria, complaints of a protruding mass, obstructive urinary symptoms, including a weak urinary stream, urethral discharge, flank pain, and hydronephrosis are symptoms related to urinary tract MMs [64].

## 4. Diagnosis

MMs presenting ‘‘ABCDE’’ features can be clinically recognized in visible regions such as the vulva, the penis, and the oral cavity [15]. However, it is important to take into account that amelanocitic lesions may be difficult to diagnose.

A careful clinical examination as well as palpation for the detection of suspicious regional or distant lymphadenopathies along with a visual inspection for detection of occult mass must be carried out in such patients.

Particular attention should be paid to cutaneous examination and careful exploration of visible mucosae due to the possibility of metastatic CM. Specialist consultations by an otolaryngologist, urologist, gynecologist, and gastroenterologist are often necessary for MM diagnosis because of the necessity to perform biopsies of suspicious lesions. Pathology examination of biopsy specimen represents the gold standard for diagnosis.

Macroscopically pigmented lesions are highly suspect for melanoma. If pigment is absent, then diagnosis may be less simple. Immunohistochemical staining positive for protein S-100, HMB-45, Melan-A, Mart-1, and tyrosinase support the diagnosis of melanoma [1].

## 5. Staging

Once diagnosis of malignancy is performed an accurate staging of the disease is mandatory in order to obtain prognostic information and to determine the further management of the patient. Usually, laboratory analyses along with instrumental tests including endoscopic studies of the head and neck region or gastrointestinal tracts, a total body computed tomography (CT) scan, positron emission tomography (PET), ultrasonography of various areas within the human body, as well as magnetic resonance imaging (MRI) are useful to complete the diagnostic path and to detect putative loco-regional and systemic extension of the disease. However, no consensus on staging MMs from various anatomic sites exists [41].

Due to the rarity of disease, the adequacy of staging criteria for providing prognostic information is not clear and many oncologists use different systems to stage MM [41]. Globally, MMs of the aero-digestive tract are staged in Italy according to AJCC staging (8° ed) and vulvar melanomas are staged following the AJCC staging criteria for CM; for the urethral, vaginal, rectal, and anal MMs, there is no consensus for staging [70].

Some clinicians prefer to utilize a simple and practice system for staging MM patients, which was proposed by Ballantine for Head and Neck MMs in 1970 [71]. This system is characterized by three stages, depending on disease extension: (I) local, (II) regional (nodal metastases), or (III) disseminated.

## 6. Management

### 6.1. Surgery

Surgery represents the mainstay for treatment of localized disease. Complete tumor removal with the pathological finding of clear margins seems to be associated with better outcomes [72,73], though with no clear benefit on overall survival (OS) [41].

Despite aggressive surgery, the occurrence of local or distant recurrences are very common with the majority of patients ultimately dying of disseminated disease. Factors associated with local recurrence include tumor size, vascular invasion, and not radical tumor resections.

Furthermore, the surgical strategy may be challenging and it should be tailored individually taking into account the tumor stage or its anatomic site, because of its potential morbidity. Overall, the poor prognosis related to the disease induces the consideration of the residual patient quality of life in any clinical decision-making in order to avoid unnecessary and harmful surgical efforts. For instance, endoscopic resections can be offered to selected patients with lower morbidity and similar local control [74].

### 6.2. Radiotherapy

Radiotherapy may be helpful for increasing local control and reducing the rates of local failure, especially when surgery achieves no negative margins or is aimed at obtaining conservative surgical management of the disease such as in anorectal MMs [75]. It is also a treatment worth considering for bulky mass or symptomatic lesions with palliative scope.

### 6.3. Systemic Therapy

Before 2010, the prognosis of patients affected by advanced CM was very poor irrespective of melanoma subtype, as few effective systemic therapies were available. In their series, Kuck et al. [7] reported a median survival ranging from 10 to 13 months in patients affected by metastatic melanoma from cutaneous, acral, uveal and unknown origin [27]. In the same report, patients affected by MM exhibited a poorer outcome with a median survival of approximately 9 months [7].

Whilst the introduction of new systemic treatment options for the management of patients affected by CM, such as immunotherapies (anti-CTLA-4 and anti-PD-1 antibodies [76]) or targeted therapies (BRAF, MEK inhibitors [77]) either alone or in combination, represented a significant breakthrough in clinical practice, the development of systemic therapies for MMs has been much slower [7].

Actually, 3 immune checkpoint inhibitors (ICIs) have been approved for treatment advanced melanoma patients: ipilimumab, a monoclonal antibody against cytotoxic T-lymphocyte-associated protein 4 (CTLA-4); the anti-programmed cell death receptor 1 (PD-1) agents pembrolizumab and nivolumab; as well as the combination of ipilimumab plus nivolumab [78]. Recently, based on results of the phase 2/3 Relativity-047 randomized trial [79], a new drug that combines nivolumab with relatlimab, opdualag, has been approved in an attempt to block both the immune checkpoint protein PD-1 and LAG-3.

Across different trials, PD-1 blockade with either nivolumab or pembrolizumab resulted in response rates ranging from 26% to 44% when used as single agents and significantly improved OS when compared with ipilimumab and dacarbazine [78,80,81,82,83,84,85]. Furthermore, a durable, sustained survival benefit can be achieved with first-line nivolumab plus ipilimumab or nivolumab alone [86].

However, CM and MMs are molecularly different tumors and most of these studies have been performed on patients affected by CM, limiting data extrapolation to MMs because of their rarity.

It was also suggested that the efficacy outcome of ICIs was poorer in MM than CM patients, but no formal perspective comparisons, to the best of our knowledge, were made between such melanoma subtypes [51,87]. Moreover, due to the rarity of MMs, knowledge about disease treatment with ICIs are limited, mostly deriving from small population studies, retrospective analyses, and case reports [26,88,89,90,91,92].

A multicenter retrospective study comparing immunotherapy (anti-PD-1 and anti-CTLA-4) versus chemotherapy (mostly dacarbazine) showed a longer median OS for patients affected by advanced MM who received ICIs (approximately 16 months) respect to those treated with chemotherapy (8.8 months) [19,93]. Furthermore, Hamid et al. reported an overall response rate (ORR) of 22% and 15% with a median progression-free survival (PFS) of approximately 2.8 months (in ipilimumab-naive or ipilimumab pre-treated patients, respectively) in a post-hoc analysis of pembrolizumab of KEYNOTE-001, -002, and -006 trials [19,94].

A large report of the efficacy and safety of an ICI in MM was reported by D’Angelo et al. in their pooled analysis, where the combination regimen nivolumab plus ipilimumab was compared with either nivolumab or ipilimumab alone [89]. This combo immunotherapy achieved a better ORR (37.1%) as compared with both single agents (23.3% and 8.3%, respectively), but showed a higher toxicity. Despite this positive result, ORR by this combination was found to be markedly inferior as compared with the ORR of 60% observed in CM patients. Furthermore, the same combo achieved 43% of ORR with a PFS of 5.8 months in a naive subgroup of MM patients from Check-Mate 067 as compared with nivolumab (ORR 30% and PFS 3 months) and ipilimumab (ORR 7% and PFS 2.6 months) [19,95].

The combo ipilimumab–nivolumab regimen appears to be a rational systemic approach for treating fit patients affected by advanced MM, though this type of treatment is burdened by important grade 3–4 toxicities (approximately 55%) [76]. Alternatively, a less toxic monotherapy with anti PDl-1 agents, such as nivolumab or pembrolizumab, may be considered for unfit patients in order to implement their treatment compliance.

A retrospective cohort study evaluated treatment efficacy of anti-PD-1 ± ipilimumab in 545 MM patients. Three hundred and forty-eight (64%) patients received anti-PD-1 whereas the remaining 197 (36%) received the anti-PD-1/ipilimumab combination. The ORR, PFS, and OS did not significantly differ by primary site (naso-oral, urogenital, anorectal, other), ethnicity/race, and treatment. Only the ORR for naso-oral primary site was numerically higher for the anti-PD-1/ipilimumab combination compared with anti-PD-1 without survival benefit. These data suggest that in MMs, the addition of ipilimumab has a poor clinical benefit over anti-PD-1 [96].

In phase 2/3 Relativity-047, 51 (7.1%) of the 714 melanoma patients were MM patients. The subgroup analysis comparing 23 cases undergoing the relatlimab–nivolumab combo toward the 28 cases undergoing nivolumab alone showed no significant differences in PFS. However, the small sample size and the rarity of MMs show that additional studies are needed to understand the efficacy of the relatlimab–nivolumab combo in patient populations that are often excluded from clinical trials [79].

Overall, it is not clear why the response rates observed to ICIs are lower in MMs with respect to CM. It has been hypothesized that this may be a result of the lower proportion of MM patients carrying high levels of PD-L1 expression (>5%), which was found in the pooled analyses, although no evidence fully supports the role of PD-L1 in predicting response to ICIs in such patients [41]. On the other hand, the lower MM mutation burden as compared to that observed in CM lesions may justify this clinical observation, whereas the presence of tumor-infiltrating lymphocytes (TILs) could be a predictor of anti PD-L1 response [41].

Recently, a phase II trial aimed to compare toripalimab versus high-dose interferon-α2b (HDI) as an adjuvant therapy for resected MM. A total of 145 patients with resected MM were randomized (1:1) to receive HDI (n = 72) or toripalimab (n = 73) for 1 year. Toripalimab showed a similar relapse-free survival and a more favorable safety profile than HDI, suggesting that toripalimab might become the better treatment option [97].

To date, limited data regarding the efficacy of neoadjuvant checkpoint inhibition for resectable MMs are available. However, the Ho et al. study, despite the small sample size (n = 36) demonstrated that the neoadjuvant anti- PD1 +/− anti-CTLA4 immunotherapy can be a feasible approach. Indeed, median event-free survival (EFS) was 9.2 months, ORR was 47%, PRR was 35%, whereas the 3-year OS rate was 55%. Interestingly, the pathologic response achievement was associated with a significant improvement in OS and EFS suggesting the need for further investigation [98].

Unlike CM, BRAF mutations are infrequent in MMs and therefore treatment with BRAF kinase inhibitors (i.e., dabrafenib or vemurafenib), mostly in combination with a MEK inhibitor (i.e., trametinib), rarely represents a therapeutic option for these patients [41]. However, all MM patients should be tested for BRAF mutations and the treatment with the combination of a BRAF inhibitor and a MEK inhibitor may be considered when activating BRAF mutations are detected [41]. Treatment with BRAF inhibitors achieved a 20% of ORR and 70% disease control rate (DCR) in a small cohort of 12 MM patients with metastatic or unresectable BRAF V600E-mutant melanoma [99].

MMs may present activating mutations in KIT. These mutations, mainly detected in exons 11 and 13, are most common in vulvovaginal and anorectal disease sites [18,19,22,46]. Tyrosine kinase inhibitors, such as imatinib or nilotinib, targeting the aberrant protein gene product were found to be able to induce a clinical response. In a multicenter phase 2 trial involving 17 MM patients harboring mutationally activated or amplified KIT, Hody et al. [100] reported a 64% of ORR among those with KIT mutations (in exon 11, 13, and 17) treated with imatinib mesylate, whereas the drug was ineffective in patients only presenting KIT amplifications. Furthermore, nilotinib exhibited similar responses as imatinib, demonstrating a clinical effect in circumstances of disease progression after imatinib [101,102,103].

Interestingly, new strategies for the systemic treatment of MMs have been recently reported [26,104,105]. These systemic approaches were developed considering the important role played by the vascular endothelial growth factor (VEGF), which is strongly expressed in MMs, on either disease progression or immunosuppression [106,107,108,109].

Sheng et al. [26,104] investigated a combination of a VEGF inhibition (axitinib) with PD-1 blockade (toripalimab) in a phase Ib trial of 33 patients suffering from metastatic MM. Among 29 treatment naïve patients, an ORR of 48.3% with a disease control rate (DCR) of 86.2% was reported. Median PFS was 7.5 months while median OS was 20.7 months. In their report, PD-L1 expression or TMB value presented no significant differences in responder versus non-responder patients, whereas gene expression profile (GEP) scores of eight selected immune-related and four angiogenesis-related genes, showed a strong correlation with clinical response [26,104]. Notably, 39.4% of patients experienced Grade 3–4 toxicities but only one patient discontinued treatment. Most common side effects were diarrhea, proteinuria, hand–foot syndrome, and hypothyroidism [104].

On the other hand, Yan et al. [105] conducted a multicenter 2:1 randomized, open-label, phase II study involving 114 naïve advanced MM patients receiving chemotherapy (carboplatin AUC 5 plus paclitaxel 175 mg/m^2^ every 4 weeks) with or without VEGF inhibition (bevacizumab 5 mg/kg every 2 weeks). The primary end point of the trial was PFS while OS was a key secondary end point [110]. The combination of carboplatin plus paclitaxel and bevacizumab was found to be more active than carboplatin plus paclitaxel. A combination with the antiangiogenic drug bevacizumab significantly increased median PFS from 3.0 to 4.8 months and median OS from 9.0 to 13.6 months [103]. Treatment appeared to be well tolerated and no significant safety signals (including no gastrointestinal perforation or hemorrhage) were reported [105,110].

This regimen could represent an alternative for the treatment of advanced MMs in an immunotherapy-ineligible or immunotherapy-failure population and further studies are warranted as suggested by the authors [105].

### 6.4. Future Therapeutic Perspectives

The different sites of origin associated with a remarkable genetic heterogeneity make MM a straightforward model to evaluate the cell biochemical changes that enhance tumor spreading. Indeed, mucosal melanoma cell lines capable of maintaining their heterogeneity following long-term cell culture and allowed to establish as two different metabolic profiles, the first characterized by the production and excretion of lactate in the extracellular microenvironment while the second, oxidative type was characterized by the uptake of lactate from the microenvironment and its use as primary carbon source, showed different phenotypic profiles in promoting cancer metastasis spreading [111]. Thus, a deep knowledge of metabolic reprogramming processes may highlight potential targets useful in interfering with the dynamic cancer progression. Another targetable metabolic pathway for MM is represented by the degradation of tryptophan to kynurenine, which plays a key role in regulating the immune response. In particular, it has been found that in tumor tissue an increase in the enzyme indoleamine 2,3-dioxygenase (IDO) that regulates the degradation of tryptophan is able to promote the development of an immunosuppressive microenvironment that can inhibit effective antitumor immunological responses [112,113]. To date, it is unknown whether in MM the IDO activity changes reflect or are able to modify the metabolism of the host, leading to serum variations in the concentration of tryptophan and kynurenine.

Interestingly, it has been observed that the surgical treatment of non-cutaneous melanoma is able to profoundly change the metabolic profile of the host. The removal of a primary melanoma of the lung re-established the level of 5-S-Cysteinyldopa within the normal range; thus, indicating how melanoma is able to condition not only the microenvironment surrounding the tumor but also to create important metabolic changes in different body matrices [114,115,116]. Considering that the metabolic profile tended to change according to the recurrence of the disease and to the treatment, the achievement of a homeostatic balance similar to normal range or its further imbalance could be considered as two different trajectories associated to different prognoses.

The cell-free microRNas (miRNAs) may play a significant role in the development and progression of MM since in MM cells it has been observed that overexpression of some miRNa, such as miR-let-7b or miR-let-7c, is able to inhibit cell growth, migration, invasion, and metastatization whereas, conversely, it seems to induce apoptosis and cell cycle shutdown after chemotherapy treatment [117]. The overexpression of these miRNAs was therefore able to reduce the recurrence of MM increasing the sensitivity to chemotherapy treatment thus leading to a lengthening DFS. In agreement with such evidence, a low expression of microRNA-23a-3p has been significantly associated with poor outcomes. Indeed, the Kaplan–Meier survival analysis of 117 MM patients showed that low mir-23a-3p expression was significantly associated with poor outcomes, with both the DFS and OS being markedly reduced. In this study, even after a multivariate analysis, the low expression of miR-23a-3p was confirmed as an independent prognostic indicator of reduced OS [118].

Finally, the recent SARS-CoV-2 pandemic allowed for the introduction of new technologies based on mRNA vaccines. This methodology has proven to be safe and effective and could be translated into the oncology field. The building of mRNAs that go inside the immune cells favoring the production of individual, specific tumor proteins able to enhance immunological responses would definitively pave the way for highly personalized therapies. Briefly, mRNA vaccines represent an attractive vector to deliver tumor antigens into dendritic cells (DCs) that are the antigen-presenting cells of our immune system [119]. The encoded proteins can be translated and processed into peptides, which can be subsequently exhibited on the DCs’ surface; thus, enhancing the immunological effects mainly through adaptive immune responses. In this context, the peptides sharing with the CD8+ T cells trigger the cytotoxic reaction while exposure to CD4+ T cells determines antibody production against the tumor cells [119]. Underlying the immune response processes turns out to be the relevant modulation of the interferon type I (IFNs) capable of initiating or adjuvating the cytotoxic activity of CD8 cells [120]. However, to date it remains controversial whether IFNs have a stimulatory or inhibitory action on CD8+ T-cell immunity since the mechanisms behind this duality are unclear.

Strategies to optimize the intracellular delivery of mRNA vaccines, allowing a more efficient antigen translation and providing potent and specific immune activations, are being investigated. Among others, lipid formulations seem to induce antigen-presenting cell maturation via the intracellular stimulator of interferon genes (STING) pathway and result in systemic cytokine expression and enhanced anti-tumor efficacy [121].

To further support such a therapeutic strategy to a wider extent, preliminary results from a study on adjuvant treatment with the personalized cancer vaccine mRNA-4157 and pembrolizumab in stage III/IV melanoma patients undergoing complete resection of cutaneous melanoma have just demonstrated a statistically significant and clinically meaningful reduction in the risk of disease recurrence or death compared to pembrolizumab monotherapy (KEYNOTE-942) [122].

## 7. Conclusions

The life expectancy of patients with MMs remains lower than that of patients with CMs since they are diagnosed at a more advanced stage and have a lower burden of point mutations and a wider number of structural chromosomal variants that make them less responsive to immunotherapy treatments. These biochemical features impose the need to identify new specific and selective druggable transcriptomics and metabolic pathways for future more efficient therapeutic applications. In this context, the application of *omics’* technologies could represent an innovative impulse to better understand the dynamic cell metabolic reprogramming that leads to cancer development and progression explaining the phenotypic heterogeneity and its biological and biochemical behavior of MMs that can contribute to improve their diagnosis and treatment.

## Data Availability

Not applicable.
